# Baicalin regulates autophagy to interfere with small intestinal acute graft-versus-host disease

**DOI:** 10.1038/s41598-022-10564-7

**Published:** 2022-04-21

**Authors:** Xiaoqi Sun, Michael Pisano, Longjin Xu, Fumou Sun, Jie Xu, Wei Zheng, Xiujuan Liu, Yanyu Zhang, Runjie Sun, Xing Cui

**Affiliations:** 1 Department of Traditional Chinese Medicine, Shandong University of Traditional Chinese Medicine, Jinan, China; 2 University of Iowa Interdisciplinary Program in Immunology, University of Iowa, 108 Calvin Hall, Iowa City, IA 52242-1396 USA; 3 Division of Hematology and Oncology, Department of Medicine, Medical College of Wisconsin Milwaukee, 8701 Watertown Plank Road, MFRC 6033, Milwaukee, WI 53226 USA; 4 Department of Osteoporosis, Center for Disease Control and Prevention of Shandong Province, Jinan, China; 5 Department of Hematology, Affiliated Hospital of Shandong University of Traditional Chinese Medicine, Jinan, China; 6 Department of Cardiovascular, Affiliated Hospital of Shandong University of Traditional Chinese Medicine, Jinan, China; 7 Center of Oncology and Hematology, Chinese Medicine/Shandong Hospital of Integrated Traditional Chinese and Western Medicine, The Second Affiliated Hospital of Shandong University of Traditional, 1 Jingba Road, Jinan, 250014 China

**Keywords:** Biochemistry, Cell biology, Immunology, Stem cells

## Abstract

Acute graft-versus-host disease (aGVHD) is the main complication of and cause of death after allogeneic hematopoietic stem cell transplantation. Baicalin can protect the small intestinal epithelial cells of rats against TNF-α-induced injury and alleviate enteritis-related diarrhea. To verify whether baicalin can protect the small intestinal mucosal barrier by regulating abnormal autophagy and interfering with intestinal aGVHD, a mouse model of aGVHD was established. CB6F1 micewere intravenously injected with a suspension of mononuclear cells derived from BALB/c donor mouse bone marrow and splenic tissue after treatment with 60Co X-rays. After treatment with different doses of baicalin for 15 days, the survival time, serum TNF-α and IL-10 levels, and autophagy markers levels in the intestine were assessed. A cell model of intestinal barrier dysfunction was also used to verify the effect of baicalin. The results showed that baicalin significantly prolonged the survival time, significantly reduced the aGVHD pathology score and clinical score by decreasing the TNF-α level with increasing the IL-10 level compared with the control. Transmission electron microscopy examination showed that baicalin treatment increased the number of autophagic vacuoles and led to the recovery of mitochondrial structures in the intestinal mucosal epithelial cells of mice and in Caco-2 cells. Western blotting results showed that baicalin treatment enhanced autophagy in vivo by regulating the AMPK/mTOR autophagy pathway. Similar results were observed in vitro in Caco-2 cells. Furthermore, the effect of baicalin was reduced after combination treatment with the autophagy inhibitor 3-methyladenine(3-MA). Baicalin can decrease the severity of small intestinal aGVHD by regulating autophagy by influencing imbalances in inflammatory cytokine levels and mucosal barrier damage, thus baicalin may have potential as a new treatment for aGVHD.

## Introduction

Acute graft-versus-host disease (aGVHD) is one of the most fatal complications that occurs in the early period after hematopoietic stem cell transplantation. Autophagy is a process by which a cell phagocytoses its own cytoplasmic proteins or organelles, and encapsulates them into vesicles that fuse with lysosomes to form autophagic lysosomes. Autophagic lysosomes then degrade their contents, which are recycled to meet metabolic needs of the cell and the renewal of certain organelles. Miten’s work showed that interference with the mTOR pathway may protect against aGVHD after bone marrow transplantation^1^. A study found that the intraperitoneal administration of metformin, which activates AMPK signaling, ameliorated the clinical severity and mortality rates of aGHVD^2^.

The molecular formula of Baicalin is C21H18O11, and baicalin was isolated from *Scutellaria baicalensis* Georgi and is broadly used as a treatment for inflammatory diseases. Baicalin has been demonstrated to exert a protective effect on IEC-6 cells (rat small intestinal epithelial cells) cells against TNF-α-induced injury^3^, and reduce serum OVA-specific sIgE levels in rats with food allergies to alleviate intestinal damage^4^. Baicalin can also regulate the NF-κB activation and modulate both autophagic and inflammatory processes, leading to an improvement in the paracellular permeability of LPS-stimulated intestinal cells^5^. In a model of human skin fibroblast cells exposed to ultraviolet B radiation, baicalin promoted the induction of autophagy through the AMPK-mTOR pathway, ameliorating radiation-induced damage^6^. Despite its potential for use as a treatment, there have been no studies on the effects of baicalin on intestinal aGVHD. Therefore, we performed experiments to explore the mechanism by which baicalin regulates autophagy in the context of intestinal aGVHD to explore its potential for use as a novel treatment for aGVHD in the clinic.

## Materials and methods

### Reagents and antibodies

Baicalin (98%), with batch number YS121121, was purchased from the Xi'an Yuansen Biotechnology Company. 3-MA and LC3-II, LC3-I, Beclin-1, P62, p-AMPK, AMPK, mTOR, and tubulin monoclonal antibodies were purchased from Affinity Biosciences (Cincinnati, OH, USA). The CCK-8 kit, RIPA lysis buffer, polycarbonate Lucifer Yellow (dextran) and the reverse transcription kit were purchased from Sparkjade (Jinan, China). Bafilomycin A1 was purchased from BioVision Incorporated (Waltham, MA, USA). The Caco2 cell line was obtained from the Chinese Academy of Sciences (Beijing, China). The Millicell system and fluorescence plate reader were obtained from Sparkjade (Jinan, China).

### Animals and experimental protocol

Mice were purchased from Beijing Weitong Lihua Experimental Animal Technology Co., Ltd. and the animal production license number was SCXK (Beijing) 2012-0001. Ten healthy SPF-grade BALB/c H-2d male mice were used as donors (weighing 18–22 g and aged 8–10 weeks),were used as donors, and 56 SPF-grade CB6F1 female mice were used as transplant recipients(weighing 18–22 g and aged 8–10 weeks) were used as transplant recipients.

Inbred BALB/c H-2d mice were sacrificed by cervical dislocation, and mononuclear cells were aseptically harvested from the bone marrow and splenic tissue aseptically. Next, CB6F1 mice were irradiated with 5.0 Gy 60Co X rays.Then, the mice were transplanted with a suspension of mononuclear cells (1 × 10^7^ bone marrow cells + 1 × 10^7^ splenic cells) harvested from donor BALB/c mice (above) by infusion via the tail vein^7^. The presence of the Y chromosome in the bone marrow of the recipient mice was used to identify cells from the donor mice and confirm the successful transplantation of donor cells into the recipient CB6F1 mice.

The mice were randomly divided into the aGVHD control and 15, 30 and 60 mg/(kg d) baicalin groups. The aGVHD control mice were administered normal saline and the treated mice were administered different doses of baicalin for 15d immediately after the model was established.

To examine the influence of baicalin-induced autophagy, we also compared treatment with 30 mg of baicalin alone to treatment with 30 mg of baicalin combined with an intraperitoneal injection of 15 mg/(kg d) 3-MA^8^ or 0.1 mg/(kg d) bafilomycin A1(Baf-A1).All the animal procedures were performed with the approval of the Animal Ethics Committee of the Affiliated Hospital of Shandong University of Traditional Chinese Medicine. All the experiments were performed in accordance with relevant guidelines and regulations.

To confirm whether baicalin acts on intestinal cells directly or through immune cells, we also established a syngeneic HCT model. CB6F1 mice were lethally irradiated with 5.0 Gy 60Co X-rays and intravenously injected via the retro-orbital route with 4 × 10^6^ bone marrow cells and 1 × 10^6^ splenocytes from CB6F1 mice. Then, the syngeneic HCT model control mice were administered normal saline, and the treated mice were administered 30 mg/(kg d) baicalin for 15 d immediately after the model was established.

### Cell line culture and transepithelial electrical resistance testing

Human Caco-2 colon carcinoma cells were cultured in Dulbecco’s modified Eagle’s minimum essential medium (DMEM, pH 7.4) (Invitrogen) supplemented with 25 mM glucose, 10% inactivated fetal bovine serum (FBS) (Lonza), 1% penicillin streptomycin (PS) and 1% nonessential amino acid solution (Invitrogen). After the cells were stimulated with TNF-α (100 ng/ml) for 21 h at 37 °C, transepithelial electrical resistance (TER) testing was performed to verify whether the cell model of intestinal barrier dysfunction had been successfully established. For the measurement of the TER, 1 × 10^5^ cells were seeded on polycarbonate Transwell inserts with a diameter of 12 mm and a pore size of 0.4 μm. The TER was recorded using the Millicell system, and resistances obtained over a blank filter were subtracted from the measured values. Once the TER values remained stable above 250 Ω cm2, the cells could be used for the permeability studies^9^. For the Lucifer yellow, or dextran assessment, permeable Transwell inserts were washed with HBSS followed by apical application of the tracer compounds. After 2 h of gentle agitation at 37 °C, the basal tracer concentration was measured using a fluorescence plate reader and then used to determine the relative sodium-to-chloride permeability according to the Goldmann-Hodgkin-Katz equation.

After the cell model was established, the cells were treated with 10, 20 or 40 μg/ml baicalin, respectively. To confirm the effect of autophagy, 20 μg/ml baicalin was applied 1.5 h after treatment with 2 mM 3-MA or 1 nM Baf-A1.

### Clinical aGVHD classification criteria

The clinical score for aGVHD was determined by observing weight loss, posture, activity, hair, skin integrity, and diarrhea on the 15th day after transplantation (Table 1). The criteria for determining aGVHD were based on work reported by Cooke et al^10^, and we added assessment of diarrhea, these criteria are listed in the following table.

**Table 1:** aGVHD clinical score and diarrhea score criteria.

	Criteria	Grade 0	Grade 1	Grade 2
aGVHD clinical score	Weight loss	< 10%	10–25%	> 25%
	Posture	Normal	Hunching noted only at rest	Severe hunching impairing movement
	Activity	Normal	Mildly to moderately decreased	Stationary unless stimulated
	Fur texture	Normal	Mild to moderate ruffling	Severe ruffling/poor grooming
	Skin integrity	Normal	Scaling of paws/tail	Obvious areas of denuded skin
Diarrhea	Diarrhea	≤ 1 time	2–3 times	≥ 4times

Individual mice from coded cages received a score of 0–2 for each criterion (maximum score of 12), as described above.

### Small intestinal mucosa histological and pathological inflammation score analysis

Small intestinal tissue was harvested from the upper 10 cm of the cecum of the mice on the 15th day. The tissue was fixed in 4% paraformaldehyde for hematoxylin and eosin (H&E) staining following standard protocols. H&E images were observed under a light microscope (× 200). Histological damage to the tissues was quantified using a pathological inflammation score (Table 2), and this score was determined by examining damage to the small intestinal tissue, crypt destruction, degree of mucosal ulceration and extent of cell infiltration (the scores for each criterion were added to obtain the pathological score of the small intestine^11^). Pathological grading was performed in a blinded manner by the same pathologist. The grading and the counting of goblet cell numbers per 0.12 mm^2^ were performed by a pathologist who was blinded to the experimental background of the tissue samples. Motic Images Advanced 3.0 software (Motic Digital Pathology, Hong Kong, China) was used to measure the intestinal villus height and crypt depth.

**Table 2:** Pathological inflammation score in the small intestine.

Small intestine	0	0.5	1	2	3	4
Villous blunting crypt	Normal	Focal and rare	Focal and mild	Diffuse and mild	Diffuse and moderate	Diffuse and severe
Crypt regeneration						
Crypt loss						
Luminal sloughing of cellular debris						
Lamina propria inflammatory cell infiltrate						
Mucosal ulceration						

### Measurement of the gene expression levels of TNF-α and IL-10

The expression of TNF-α and IL-10 was measured using quantitative reverse transcription polymerase chain reaction (RT–PCR). Serum was collected and quickly frozen for subsequent mRNA extraction. Total RNA was extracted using the guanidine isothiocyanate-phenol–chloroform method. Extracted RNA was reverse-transcribed using the Sparkjade reverse transcription kit and the resulting complementary DNA was analyzed to measure the expression of the target molecules using the LightCycler 480 real-time PCR system. The primer sequences are provided in Table 3. The resulting gene expression levels of the target molecules were normalized to the expression level of GAPDH.

**Table 3:** PCR primer sequences.

Gene	Forward	Reverse
GAPDH	5′-CAA CTT TGT CAA GCT CAT TTC C-3′	5′-GGT CCA GGG TTT CTT ACT CC-3′
TNF-α	5′-CAT GCA CCA CCA TCA AGG AC-3′	3′-GGC CTG AGA TCT TAT CCA GCC-3′
IL-10	5′-CTA TGC TGC CTG CTC TTA CTG-3′	5′-AGC AGT ATG TTG TCC AGC TG-3′

### ELISA test analysis

The serum levels of the inflammatory cytokines interleukin-10 (IL-10) and tumor necrosis factor-α (TNF-α) were measured by an ELISA kit (Hengyuan, Shanghai, China). The test samples and the standard samples were added to the corresponding ELISA plate and cultured. After the chromogenic antibody was added to the ELISA plates, the termination solution was added to terminate the reaction. The OD value at 450 nm was measured with a microplate reader (Thermo Fisher Scientific, Rockford, IL, USA).

### Western blotting analysis

Small intestinal tissue samples or Caco-2 cells were lysed using RIPA lysis buffer and total protein was collected. Next 20 μg of each protein sample was loaded onto 10% SDS–PAGE gels, and the protein was concentrated at 80 V for 20 min and separated at 120 V for ~ 1 h. After the electrophoresis was stopped, the samples were transferred to PVDF membranes at 110 V at 4 °C following the wet blotting method protocol. Each immunoblot was blocked with 5% nonfat milk in TBST for 2 h and then incubated with primary antibodies against LC3, P62, Beclin-1, AMPK, mTOR or β-tubulin at 4 °C overnight. The membranes were washed 3 times and then incubated with diluted secondary antibodies for 2 h. After the secondary antibody solution was completely removed, a PTG ECL chemiluminescence detection kit was used to develop the blot. Then the blot was transferred to an imaging machine for exposure and analysis. The AMPK, mTOR and β-tubulin blot images in Figs. 2 and 4 are from the same gel image. because other bands on some blots are not shown in this manuscript, some full length images were not submitted in the supplementary files to avoid confusion.

### Immunohistochemistry (IHC) analysis

Tissues of the small intestine (2–3 cm) were harvested, fixed with 10% formaldehyde, decolorized, cleared, embedded in paraffin and sectioned. Antigen retrieval was performed for 20 min in a pressure cooker. The sections were cooled, washed with PBS, incubated with a 3% H_2_O_2_ solution for 10 min and washed with PBS again. The tissue sections were incubated with goat serum for 20 min, washed to remove the serum, incubated with primary antibodies against AMPK and LC3 at 37 °C for 1 h, washed with PBS, incubated with biotin-labeled secondary antibodies at room temperature for 30 min and washed again with PBS. Finally, the sections were incubated in DAB substrate solution for 5 min. After being washed thoroughly with tap water, the sections were counterstained with hematoxylin, dehydrated in absolute alcohol, cleared in xylene, and subjected to microscopy. The expression of proteins was quantified using an image analysis and measuring system (Image-Pro Plus 6.0). The mean area and mean integrated optical density (mean IOD) of the expression of these proteins were calculated.

### Immunofluorescence staining of LC3 in vivo and in vitro

Paraffin-embedded tissue sections of small intestine tissues or Caco-2 cells were incubated with primary antibodies at 4 °C for 24 h. Then, goat anti-rabbit Cy3-conjugated red fluorescent secondary antibodies were applied and incubated for 60 min at 37 °C. The samples were fixed with mounting medium supplemented with 4′,6-diamidino-2-phenylindole (DAPI) to stain the nucleus. Images were immediately captured with a fluorescence microscope. Quantification of LC3 puncta was performed using ImageJ software^12^.

### Detection of intestinal permeability

Fluorescein isothiocyanate dextran (FITC-dextran) measure was used to determine determination of intestinal permeability. For this assay, 0.5 mL of 25 mg/mL FITC-dextran (4 kDa, Sigma, St. Louis, MO) was fed to the mice, and 50 µL of blood was collected 3 h later. Then the FITC-dextran level was measured by a fluorospectrometer (NanoDrop 3300; Thermo Scientific, Wilmington, DE).

### Transmission electron microscopy (TEM)

Autophagic vesicles were observed by TEM (Hitachi, HT7700). Mouse small intestinal tissues were cut into small fragments (1 mm^3^) before fixation. Treated cells were fixed with 2.5% glutaraldehyde and sodium pyruvate for 24 h, postfixed with 2% OsO_4_ for 2 h, and then dehydrated. The fixed samples were subsequently cut into thin Sections (60 nm) with a diamond knife. After fixation, small intestine tissues were treated with 1% osmic acid for 2 h, dehydrated with increasing concentrations of ethanol, stained with uranyl acetate for 20 min and then incubated with lead citrate for 15 min. After the samples were washed and dried, images were obtained using a Philips Tecnai 10 transmission electron microscope (FEI, Hillsboro, OR, USA).

### Statistical analysis

The experimental results were analyzed using SPSS Version 19.0 (SPSS Inc., Chicago, IL, USA) and GraphPad Prism 8.0 (GraphPad Software, San Diego, CA, USA). The normal distribution of the data in each group was confirmed prior to the following analysis. The results are expressed as the mean ± standard deviation. One-way ANOVA was used to test the significance of the differences among groups, followed by Scheffe’s modified F test for multiple comparisons. *P* < 0.05 indicated a statistically significant difference.

### Ethics approval and consent to participate

All the experiments were conducted in compliance with the ARRIVE guidelines. We confirm that animal care and experimental procedures were carried out in accordance with the guidelines of the Animal Ethics Committee of the Affiliated Hospital of Shandong University of Traditional Chinese Medicine. The reference number is AWE-2019-037.

## Results

### Effect of baicalin treatment on the recovery of intestinal aGVHD in mice

**Figure 1:**
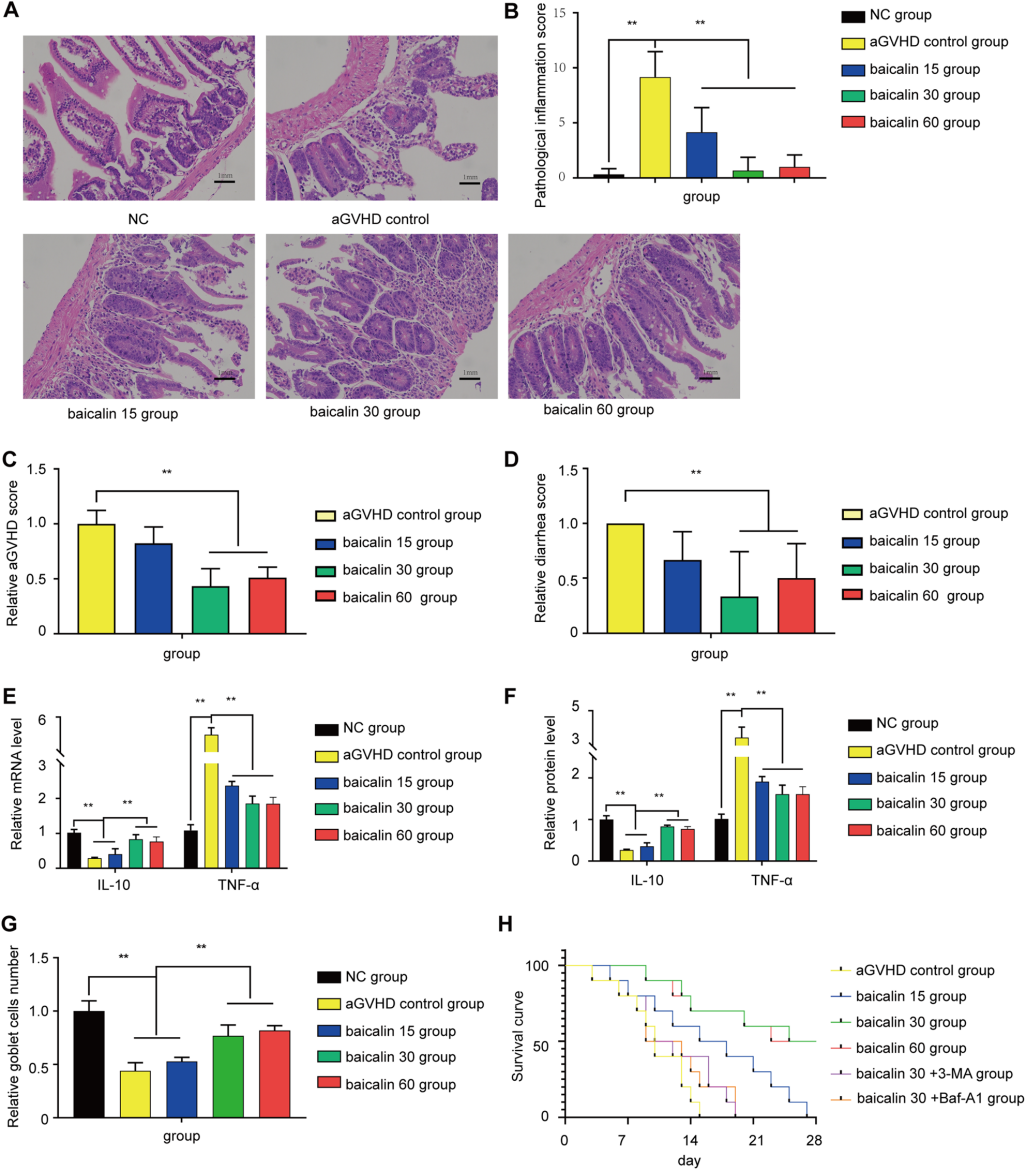
Mouse survival curve and small intestinal pathology. There were 6 mice in the normal control group, and the other groups had 10 mice in each group. (**A**) Representative intestinal histology from each experimental group on the 15th day (H&E staining, 200 ×). (**B**) Analysis of the mouse small intestine pathological scoreon the 15th day. (**C**) Clinical aGVHD score of each experimental group on the 15th day. (**D**) Diarrhea score of each group on the 15th day. € mRNA Levels of the inflammatory factors IL-10 and TNF-α in the experimental group on the 15th day. (**F**) Protein levels of the inflammatory factors IL-10 and TNF-α in the experimental group on the 15th day. (**G**) Goblet cell number in different groups on the 15th day. (**H**) Survival curve of mice of the experimental groups of mice. The data are presented as the mean ± SD. Every experiment was repeated in triplicate. **P < 0.01 compared with the aGVHD control group.

First, the establishment of the intestinal aGVHD model was verified by histological analysis of the small intestinal mucosa (Fig. 1A,B), aGVHD scoring(Fig. 1C) and diarrhea scoring (Fig. 1D). The IL-10 and TNF-α mRNA and protein expression levels (Fig. 1E,F) were also assessed to confirm the establishment of the intestinal aGVHD model. Heavy inflammatory cell infiltration was observed in the intestinal mucosa in the aGVHD control group, which is consistent with the pathological characteristics of intestinal aGVHD (Fig. 1A,B). After treatment with 30 or 60 mg/(kg d) baicalin, recovery of the small intestinal mucosal epithelial structure was indicated by multiple observations, including a decrease in inflammatory cell infiltration, an increase in the regularity of glandular cell arrangement, an increase in the number of goblet cells (Fig. 1G), a decrease in the shedding of cellular debris, and a decrease in mucosal epithelial necrosis. The pathological inflammation scores in the 30 and 60 mg/(kg d) dose groups were significantly lower than those in the aGVHD control and 15 mg/(kg d) dose groups (Fig. 1B). TNF-α expression was significantly decreased and IL-10 expression was significantly increased in the 30 and 60 mg/(kg d) dose groups compared with the aGVHD control group (*P* < 0.01,Fig. 1E).

All the mice in the aGVHD control group died by day 15 and all those in the low dose group died by day 27, whereas 50% of the mice in the 30 and 60 mg/(kg d) dose groups survived beyond day 28. When 30 or 60 mg/(kg d) baicalin was administered to the mice, a significant increase (*P* < 0.001) in overall durable survival was observed, with a significant difference in the median survival time between these treatment groups and the aGVHD control groups (27 or 26.5 d vs. 10 d) (*P* < 0.001). In contrast, the median survival time decreased to 11 d after treatment with 30 mg/(kg d) baicalin and 3-MA or Baf-A1 (Fig. 1H). These data demonstrate that treatment with 30 and 60 mg/(kg d) baicalin effectively reduces morbidity and mortality by improving intestinal mucosal injury in a mouse model of aGVHD.

### Baicalin affects intestinal aGVHD via autophagy in mice

**Figure 2:**
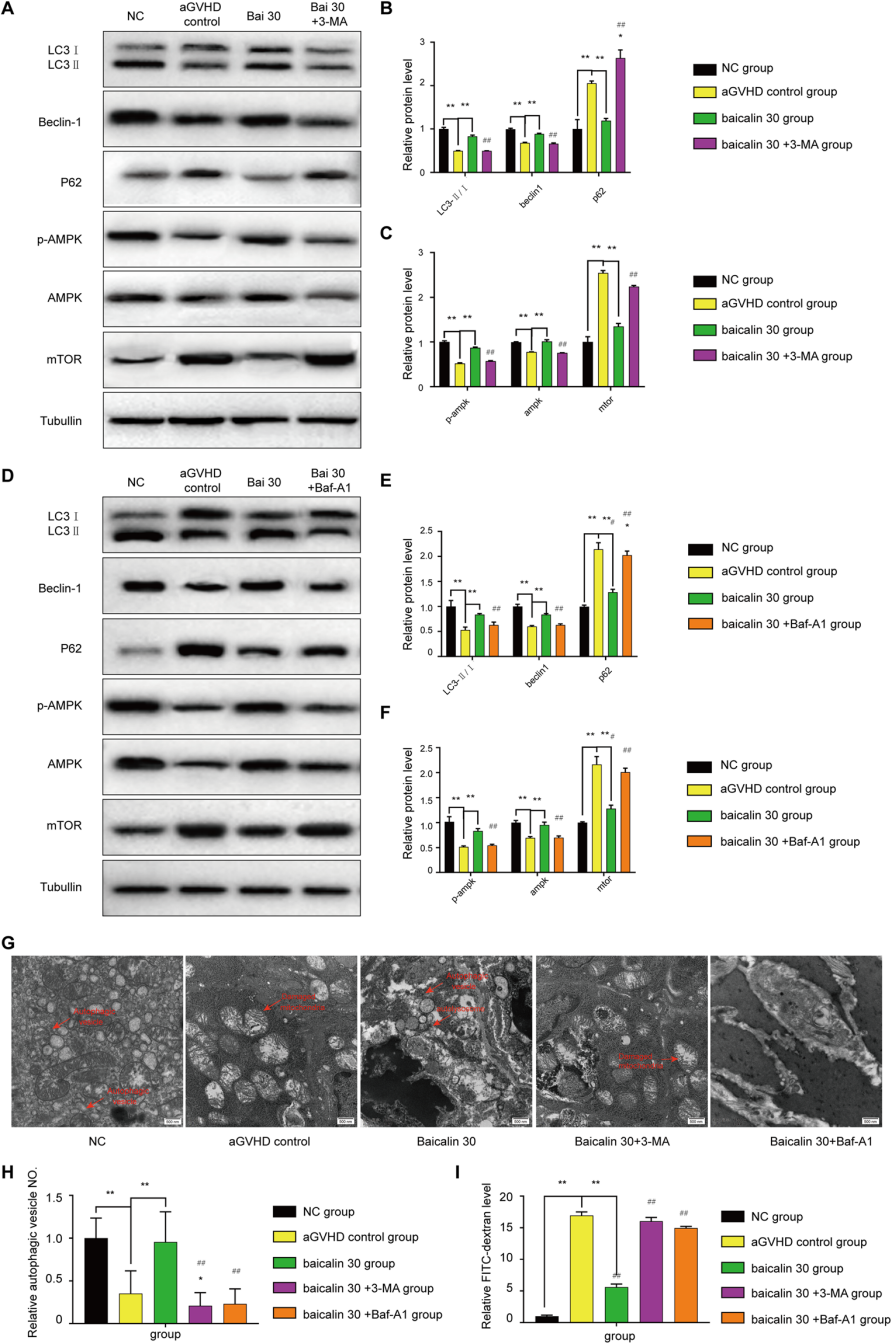
Autophagy level. On the 15th day, samples were collected for testing. (**A**) To confirm that the effect of baicalin is mediated by regulating autophagy, 3-MA was used with baicalin treatment, and the levels of the autophagy-related proteins LC3II/I, Beclin1, P62, p-AMPK, AMPK, and mTOR were measured by Western blotting. (**B**) Analysis of LC3II/I, Beclin1 and P62 protein levels. (**C**): Analysis of the p-AMPK, AMPK, and mTOR levels. (**D**) Measurement of the levels of the autophagy-related proteins LC3II/I, Beclin1, P62, p-AMPK, AMPK, and mTOR by Western blotting after treatment with or without Baf-A1 to inhibit autophagy. (**E**) Analysis of LC3II/I, Beclin1 and P62 protein levels. (**F**) Analysis of the p-AMPK, AMPK, and mTOR levels. (**G**) Autophagic vesicles observed by TEM. (**H**) Analysis of autophagic vesicle numbers in each experimental group. (**I**) Analysis of FITC-dextran levels. All the blots are representative cropped images and every set was processed simultaenously under similar conditions. Representative original blots with cropped demarcations (**A**) are provided in Supplementary Fig. 1. Each experiment was repeated three times.*P < 0.05 and **P < 0.01 compared with the aGVHD control group. ##*P* < 0.01 compared with the 30 mg/(kg d) baicilin group.

To determine the mechanism of action of baicalin, the level of mTOR-mediated autophagy after was investigated treatment with 30 mg/(kg d) baicalin. Specifically, the AMPK autophagy pathway was activated, as indicated by increased levels of LC3II/I, Beclin1, and p-ampk and decreased levels of P62 and mTOR (*P* < 0.01), (Fig. 2A–E). After autophagy was inhibited with 3-MA or Baf-A1, the baicalin-induced increase in autophagy-related protein expression levels was reversed, and the levels of these proteins reached the same levels as those in the aGVHD control group. Additionally, there were more biphasic membrane autophagosomes and mitochondria with normal structures in the baicalin group than in the control group, the baicalin 30 plus 3-MA groups or the baicalin 30 plus Baf-A1 group (Fig. 2.G, H). There was no significant difference between the baicalin + 3-MA group or baicalin + Baf-A1 group and the aGVHD control group (Fig. 2A–G). This finding indicates that autophagy mediates the effect of baicalin on intestinal aGVHD. The results of FITC-dextran measurement showed that baicalin alleviated the intestinal permeability of the mice with aGVHD (Fig. 2I).

**Figure 3:**
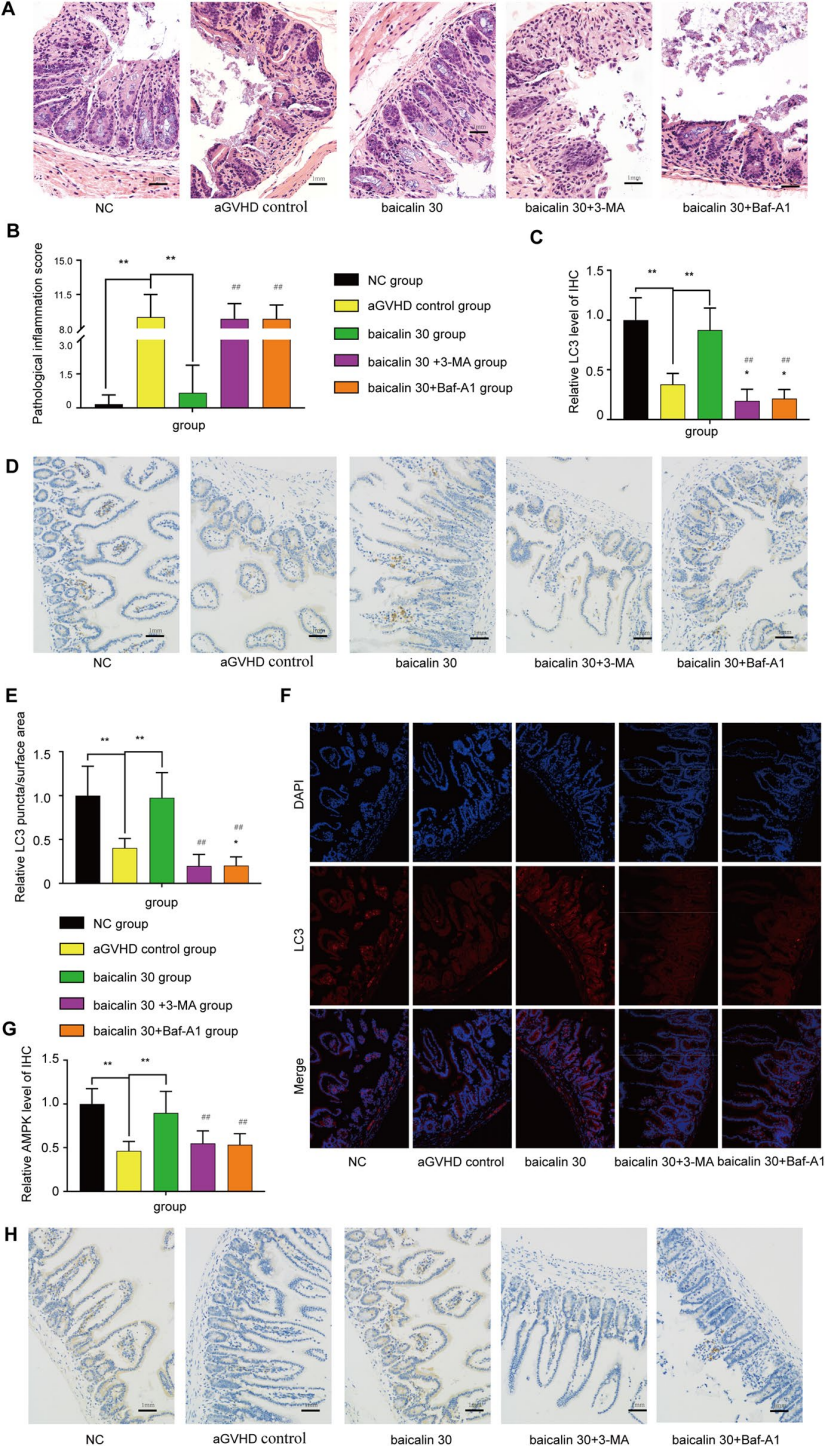
Pathology, IHC and LC3 levels of the small intestine. On the 15th day, samples were collected for testing. (**A**) Representative intestinal histological images from each experimental group (H&E staining, 200 ×). (**B**) Mouse small intestine pathological score. (**C**) Assessment of LC3 levels. (**D**) IHC staining for LC3 in different groups. (**E**) Assessment of immunofluorescence staining for LC3. (**F**) Immunofluorescence staining for LC3 in different groups. (**G**) Assessment of AMPK levels. (**H**) IHC staining for AMPK in each group. Each experiment was repeated three times. Note: *P < 0.05 and **P < 0.01 compared with the aGVHD control group. ^##^*P* < 0.01 compared with the 30 mg/(kg d) baicalin group.

Figure 3A and B show that inhibiting autophagy with 3-MA or Baf-A1 reversed the beneficial effect of baicalin treatment on aGVHD-related morbidity. The mice administered baicalin + 3-MA or baicalin + Baf-A1 exhibited far more damage to their small intestinal mucosal epithelial structure, a higher level of inflammatory cell infiltration, abnormally arranged glandular cells, fewer goblet cells, and more mucosal epithelium shedding and necrosis than mice administered baicalin alone (*P* < 0.01) (Fig. 3A,B). The level of LC3 was assessed by IHC (Fig. 3C,D) and immunofluorescence staining (Fig. 3E,F). The results showed that the LC3 level in the small intestinal tissues in the baicalin group was improved compared with that in the baicalin + 3-MA group, baicalin + Baf-A1 group and aGVHD control group. Based on the protein levels observed by Western blotting (Fig. 2A,C) and IHC (Fig. 3G,H), AMPK/mTOR induced autophagy should be an effective target of baicalin in intestinal aGVHD.

### Baicalin can improve the intestinal mucosal barrier by enhancing autophagy in vitro

**Figure 4:**
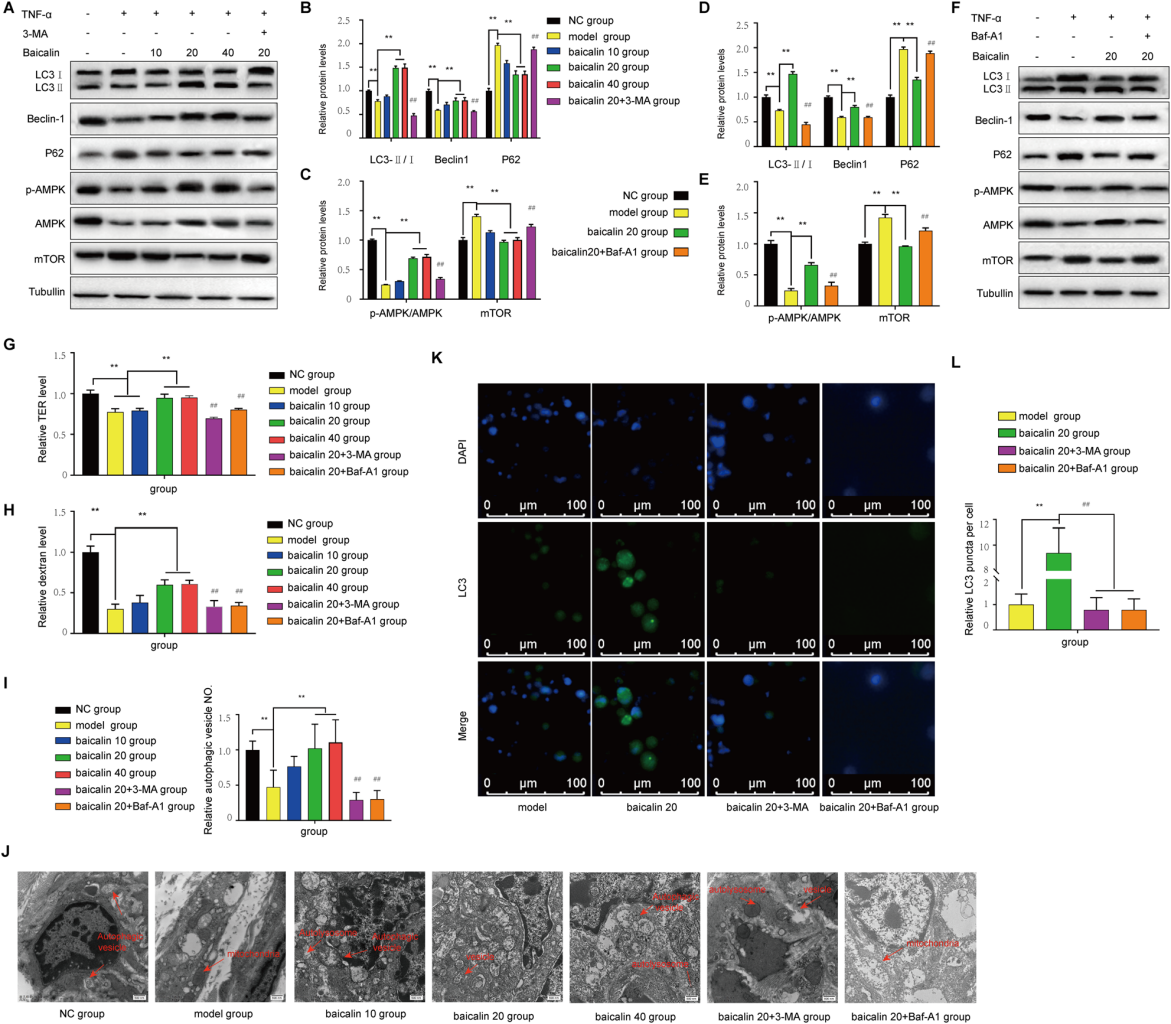
Caco2 cell experiment. (**A**) Measurement of the levels of the autophagy-related proteins LC3II/I, Beclin1, P62, p-AMPK, AMPK, and mTOR by Western blotting after treatment with or without 3-MA. (**B**) Analysis of the LC3II/I, Beclin1 and P62 protein levels. (**C**) Analysis of the p-AMPK, AMPK, and mTOR levels. (**D**) Analysis of the LC3II/I, Beclin1 and P62 protein levels after treatment with or without Baf-A1 to inhibit autophagy. (**E**) Analysis of the p-AMPK, AMPK, and mTOR levels. (**F**) Measurement of the levels of the autophagy-related proteins LC3II/I, Beclin1, P62, p-AMPK, AMPK, and mTOR by Western blotting after treatment with or without Baf-A1. (**G**) Measurement of the permeability of Caco-2 cells. (**H**) Assessment of the dextran level in each group. (**I**) Assessment of autophagic vesicle numbers. (**J**) Observation of autophagic vesicles by TEM. (**K**) Immunofluorescence staining for LC3 in different groups. (**L**) Assessment of immunofluorescence staining for LC3. All the blots are representative cropped images and every set was processed simultaneously under similar conditions. Representative original blots with cropped demarcations (**A**) are provided in Supplementary Fig. 2. Every experiment was repeated in triplicate. *P < 0.05 and **P < 0.01 compared with the model group. ^##^*P* < 0.01 compared with the 20 mg/(kg d) baicilin group.

After Caco-2 cells were treated with by TNF-α, the effect of baicalin was investigated by TER determination, Western blotting and immunofluorescence staining. We found a significant increase in the LC3II/I ratio (Fig. 4A,B,D,F) in the baicalin group. These results are similar to those found in vivo, which indicated that baicalin can promote autophagy by enhancing AMPK pathway activity and suppressing mTOR activity in Caco2 cells (Fig. 4A,C,E,F). The establishment of the intestinal barrier dysfunction model was confirmed by the TER test, and intestinal barrier dysfunction was alleviated by baicalin (Fig. 4G,H). The dextran data demonstrated that permeability increased after the cell model was established (Fig. 4H). The number of autophagic vesicles also increased (Fig. 4I,J) in the baicalin group, with increases in the number of LC3-positive foci (Fig. 4K,L), these results indicate that the autophagy level was improved. It was also found that the mitochondrial structure was restored in the baicalin-treated group (Fig. 4J). When an autophagy inhibitor was added to the baicalin treatment group, the therapeutic effect of baicalin was decreased, which was accompanied by a decrease in the autophagy level. Thus, the mechanism by which baicalin stimulates autophagy was verified again in vitro.

### Baicalin acts directly on intestinal cells

**Figure 5:**
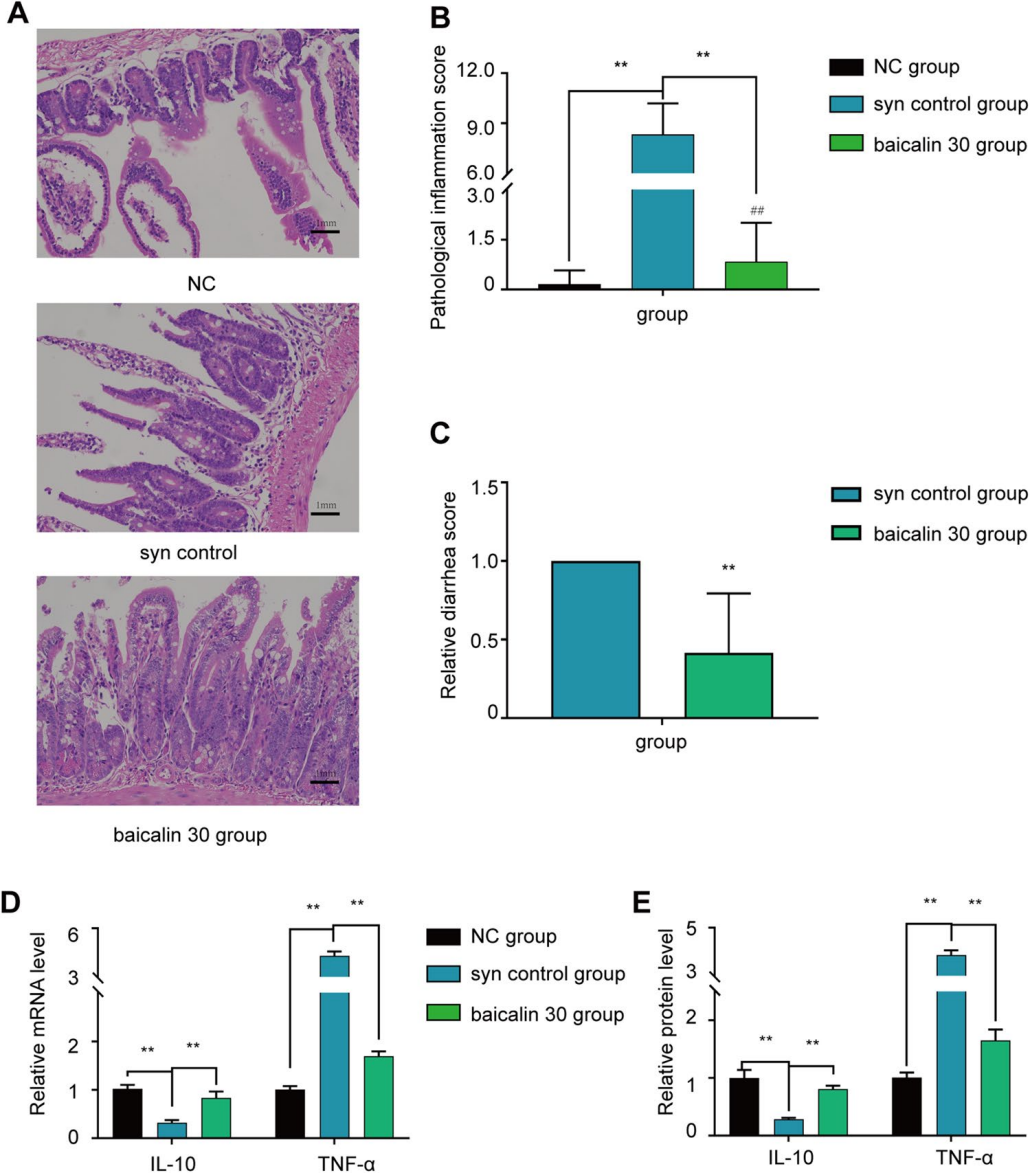
Mouse small intestinal pathology and inflammatory factor levels. There were 6 mice in the normal control group, and the other groups had 10 mice in each group. (**A**) Representative intestinal histological images of each experimental group on the 15th day (H&E staining, 200×). (**B**) Analysis of the mouse small intestine pathological score on the 15th day. (**C**) Diarrhea score of each group on the 15th day. (**D**) mRNA levels of the inflammatory factors IL-10 and TNF-α in the experimental group on the 15th day. (**E**) Protein levels of the inflammatory factors IL-10 and TNF-α in the experimental group on the 15th day. The data are presented as the mean ± SD. Every experiment was repeated in triplicate. **P < 0.01 compared with the aGVHD control group.

We also performed an experiment in a syngeneic HCT model to confirm that baicalin acts directly on intestinal cells. Compared with that in the syngeneic HCT model, the small intestinal mucosal epithelial structure recovered after treatment with 30 mg/(kg d) baicalin (Fig. 5A), and this effect was accompanied by a decrease in inflammatory cell infiltration and an increase in the regularity of glandular cell arrangement. The pathological inflammation scores and diarrhea scores in the baicalin group were significantly lower than those in the model control (Fig. 5B,C). TNF-α and IL-10 expression was the same as that observed in the aGVHD baicalin group (*P* < 0.01, Fig. 5D,E).

## Discussion

Intestinal aGVHD is one of the most common graft-versus-host reactions after transplantation. The intestinal symptoms of patients with aGVHD after transplantation are refractory diarrhea, serious colic, hematochezia and intestinal obstruction, and these symptoms can affect the success of implantation and be life-threatening. Some studies that have shown that the occurrence of intestinal aGVHD is closely related to inflammatory cell infiltration and other factors^13^. One currently recognized key factor is the pretreatment regimen which leads to cytokine storm-mediated inflammation and subsequent low immunity^14^. TNF-α and IL-10 have been recognized as biomarkers related to aGVHD^15^, and a study by Liu confirmed that the correlation between diarrhea and aGVHD was related to the serum levels of TNF-α^16^. In our study, the mRNA levels of TNF-α and IL-10 changed significantly after baicalin administration. Mucositis and infection^14^ can trigger aGVHD via destruction of the intestinal mucosal barrier. Therefore, protecting the intestinal mucosal barrier is key for treating aGVHD.

Autophagy is an important factor in maintaining gastrointestinal homeostasis^17^, and its absence is related to gut inflammation^18^. Intestinal epithelial cells from animals that lack a protein that inhibits apoptosis showed reduced levels of the autophagy-related protein LC3 after allogeneic bone marrow transplantation^19^. In the initial treatment of standard risk aGVHD, treatment with the mTOR inhibitor rapamycin resulted in similar complete/partial remission rates as treatment with prednisone on day 28. Rapamycin can change the expression of autophagy-related proteins by inhibiting mTOR and promoting autophagy^20,21^, which is similar to the effect of baicalin. Our study shows that baicalin-induced autophagy reduced the clinical aGVHD score in mice, mirroring these results. Another study showed that baicalin can induce autophagy through the PI3K/Akt/mTOR pathway, resulting in an anti-inflammatory effect^22^, which was confirmed in this study.

In our experiments, abnormal mRNA levels of IL-10 and TNF-α, which are inflammatory factors whose expression is often dysregulated in aGVHD, were corrected by treatment with baicalin. This effect was most notable in the 30 mg/(kg d) group. Additionally, after treatment with baicalin, the viability of the mice improved and the clinical aGVHD and pathological scores of the small intestine decreased. These results showed that baicalin had some ability to reduce the pathological features of intestinal aGVHD. Next we postulated that the mechanism by which baicalin affected aGVHD was by regulating the imbalance in autophagy observed in intestinal aGVHD, and we verified this by examining AMPK/mTOR signaling activity after baicalin treatment. Baicalin can increase the expression of autophagy related proteins such as LC3-II/I and Beclin 1, to increase the production of autophagic vesicles, and it can increase the levels of LC3 to promote the autophagy of cellular debris and other substances, improve the function of mitochondria, and reduce the apoptosis and abscission of intestinal epithelial cells. Some studies have suggested that autophagy activation may attenuate intestinal mucosal barrier dysfunction by preventing and reducing oxidative stress^23,24^. Our experimental results show that low levels of autophagy are associated with greater difficulty in improving Caco-2 cell permeability and that with increased levels of TNF in aGVHD mice, leads to destruction of the intestinal mucosal barrier. To confirm whether baicalin acts directly on intestinal cells, rather than through immune cells, we also established a syngeneic HCT model to investigate the effect of baicalin on protecting the intestinal mucosal barrier.

To confirm this mechanism, 3-MA and Baf-A1, which are inhibitors of autophagy, were used. The data showed that, after the inhibition of autophagy with 3-MA or Baf-A1, the effects of baicalin were reversed in vivo. Some studies have found that 3-MA can inhibit Caco-2 cell activity by inhibiting autophagy^25^. For this reason, we examined Caco-2 cells treated with 3-MA or Baf-A1 with or without baicalin.Baicalin (30 and 60 mg/(kg·d)) was found to improve intestinal barrier dysfunction by increasing the relative TER level of Caco-2 cells. Then 20 μg/ml baicalin was combined with 3-MA or Baf-A1 for the further study. We found that the effect of baicalin was weakened after the application of 3-MA or Baf-A1, providing further evidence that baicalin reduces the pathological characteristics of aGVHD by regulating autophagy.

This study provides strong evidence that baicalin can protect the intestinal mucosal barrier by promoting autophagy, reducing the death of intestinal mucosal epithelial cells, interfering with the progression of intestinal aGVHD. Our next experiments will focus on the effect of baicalin on Treg and Th17 cells in our aGVHD model to further elucidate the mechanism by which baicalin protects the intestinal mucosal barrier via immunomodulation.

## Conclusion

Based on the results described above, the following conclusions can be drawn: baicalin can interfere with intestinal aGVHD by regulating autophagy-related protein expression via the AMPK/mTOR pathway, baicalin increases autophagic vesicle production and restores mitochondrial morphology, and the effects of baicalin are weakened by inhibiting autophagy using 3-MA. Therefore, baicalin can protect the intestinal mucosal barrier and interfere with the progression of aGVHD.

## Supplementary Information

Supplementary Figures.

## Data Availability

The datasets supporting the conclusions of this article are included within the article. The datasets used and/or analyzed during the current study are available from the corresponding author upon reasonable request.
