# Supplementary material for: Baicalin regulates autophagy to interfere with small intestinal acute graft-versus-host disease
Source: Sci Rep. 2022 Apr 21;12:6551. doi: 10.1038/s41598-022-10564-7 (PMC9023573; doi:10.1038/s41598-022-10564-7)
Supplement: Supplementary file 1 — Supplementary Figures. [file 41598_2022_10564_MOESM1_ESM.docx]

**Baicalin regulates autophagy to interfere with small intestinal acute graft-versus-host disease**

Xiaoqi Sun1, Michael Pisano2,3, Longjin Xu4, Fumou Sun3, Jie Xu5, Wei Zheng5, Xiujuan Liu6, Yanyu Zhang1, Runjie Sun1, Xing Cui5*

1. Department of Traditional Chinese Medicine, Shandong University of Traditional Chinese Medicine, Jinan, China; 2. University of Iowa Interdisciplinary Program in Immunology, University of Iowa, 108 Calvin Hall, Iowa City, IA 52242-1396,USA3. Division of Hematology & Oncology, Department of Medicine, Medical College of Wisconsin Milwaukee, MFRC 6033, 8701 Watertown Plank Road, Milwaukee, WI 53226, USA4. Department of Osteoporosis, Center for Disease Control and Prevention of Shandong Province, Jinan, China5.Department of Hematology, Affiliated Hospital of Shandong University of Traditional Chinese Medicine, Jinan, China6. Department of Cardiovascular, Affiliated Hospital of Shandong University of Traditional Chinese Medicine, Jinan, China

^*^**Corresponding Author**

Dr. Xing Cui

Department of Hematology

Affiliated Hospital of Shandong University of Traditional Chinese Medicine

16369 Jingshi Road, Jinan 250014, China.

Email: [cdz45@foxmail.com](mailto:cdz45@foxmail.com)


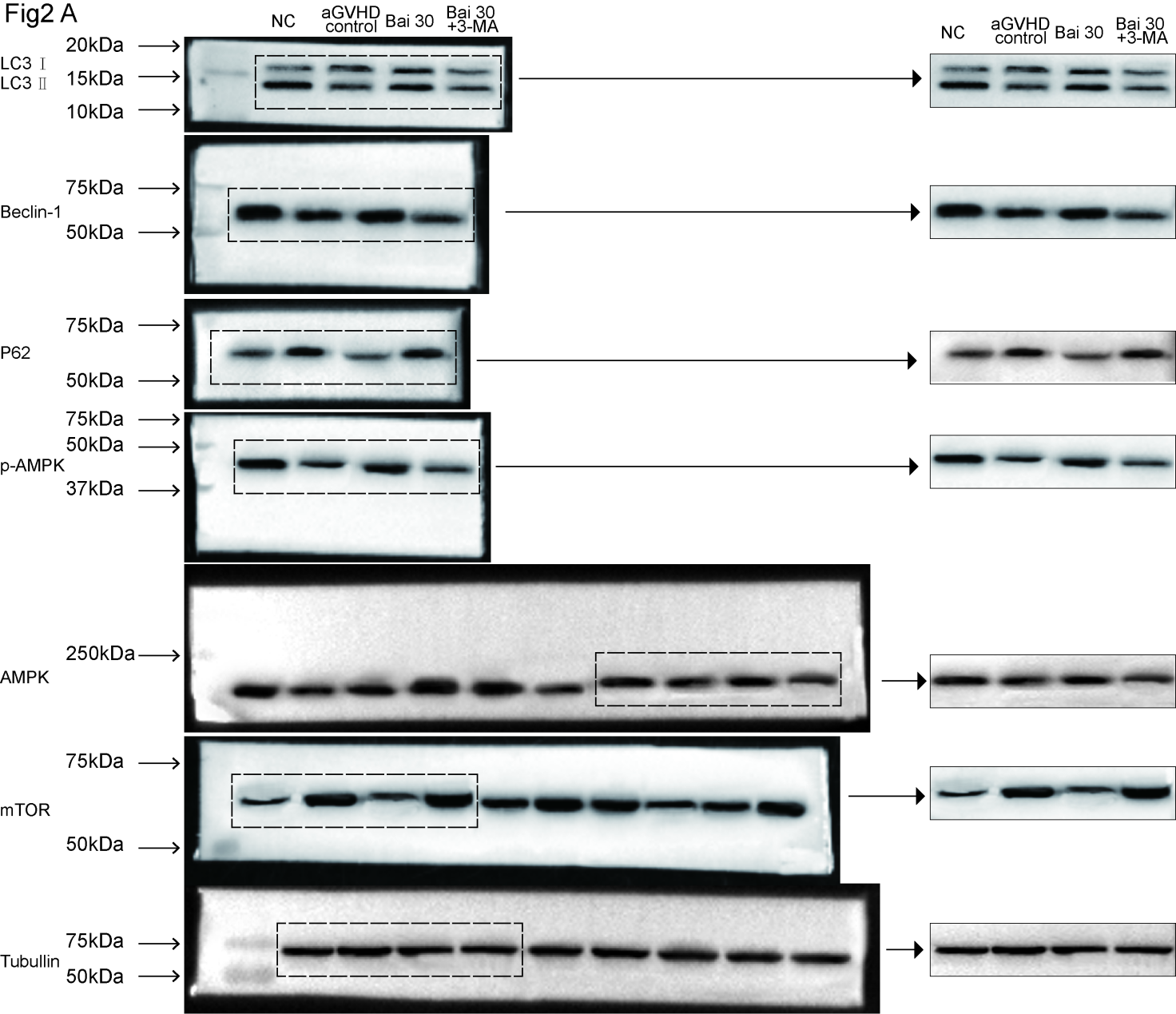


Supplementary Figure 1


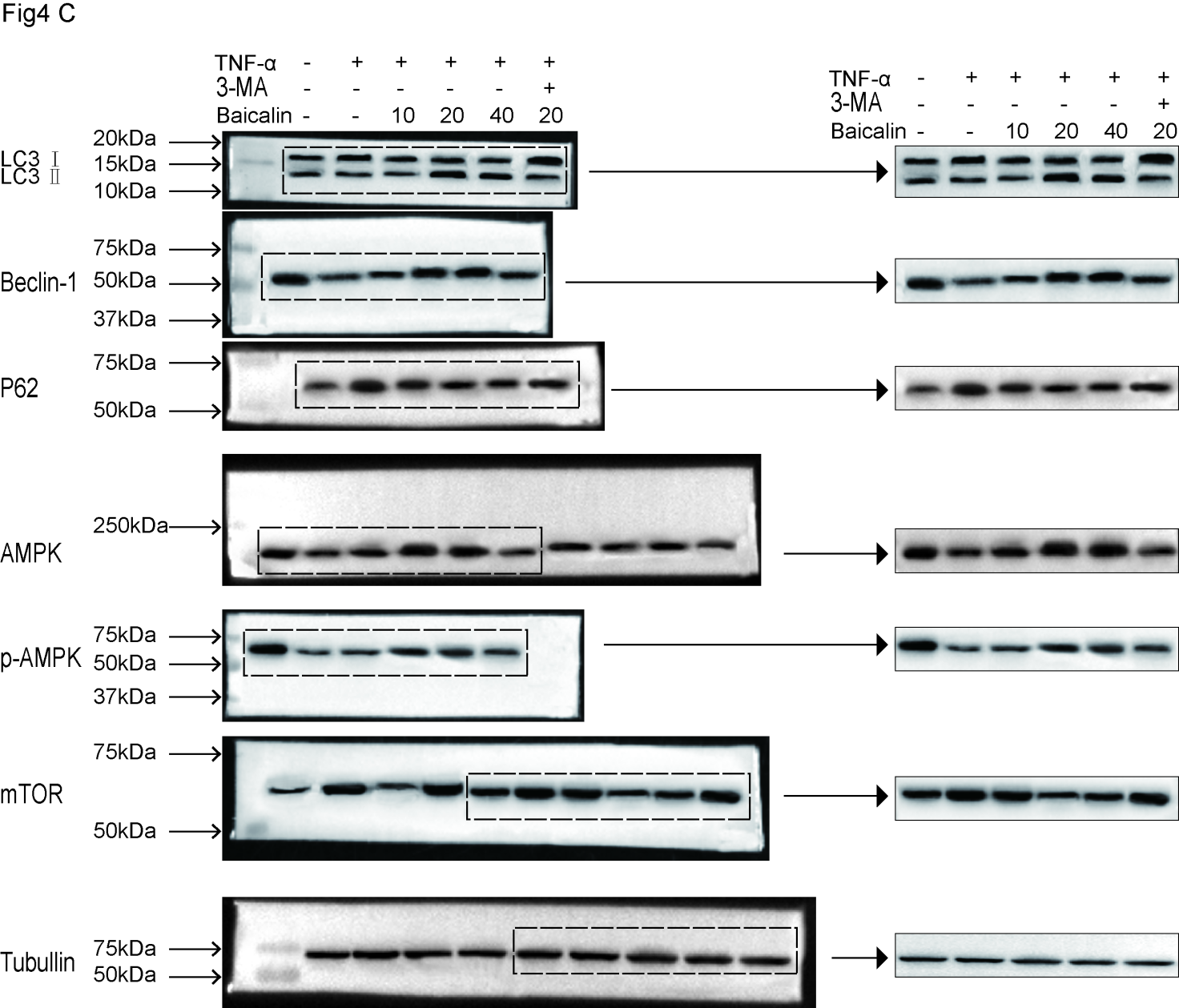


Supplementary Figure 2
